# Morphological organization of point-to-point transport in complex networks

**DOI:** 10.1038/s41598-019-44701-6

**Published:** 2019-06-06

**Authors:** Min-Yeong Kang, Geoffroy Berthelot, Liubov Tupikina, Christos Nicolaides, Jean-Francois Colonna, Bernard Sapoval, Denis S. Grebenkov

**Affiliations:** 1 Laboratoire de Physique de la Matière Condensée (UMR 7643), CNRS – Ecole Polytechnique, IP Paris, 91128 Palaiseau, France; 2 Centre de Mathématiques Appliquées, CNRS – Ecole Polytechnique, IP Paris, 91128 Palaiseau, France; 3 Research Laboratory for Interdisciplinary Studies (RELAIS), 75012 Paris, France; 4 Institut National du Sport, de l’Expertise et de la Performance (INSEP), 75012 Paris, France; 5 The Center for Research and Interdisciplinarity (CRI), University Paris Descartes, INSERM, Paris, France; 6 Department of Business and Public Administration, University of Cyprus, 1 Panepistimiou Av., 2109 Aglantzia, Nicosia Cyprus; 7 MIT Sloan School of Management, 100 Main Street, Cambridge, MA 02142 USA

**Keywords:** Complex networks, Statistical physics

## Abstract

We investigate the structural organization of the point-to-point electric, diffusive or hydraulic transport in complex scale-free networks. The random choice of two nodes, a source and a drain, to which a potential difference is applied, selects two tree-like structures, one emerging from the source and the other converging to the drain. These trees merge into a large cluster of the remaining nodes that is found to be quasi-equipotential and thus presents almost no resistance to transport. Such a global “tree-cluster-tree” structure is universal and leads to a power law decay of the currents distribution. Its exponent, −2, is determined by the multiplicative decrease of currents at successive branching points of a tree and is found to be independent of the network connectivity degree and resistance distribution.

## Introduction

Transport processes in complex networks play a crucial role in our lives, with examples ranging from transportation networks (e.g. airflight/train connections, international highways, and city transport), electricity distribution networks, microelectronic devices to news propagation and spreading of behaviors in social networks^1–11,12^. In engineering and biological applications, transport is often electric, hydrodynamic, or diffusive^13–16^. During the past two decades, a particular attention has been paid to random networks which can capture major structural properties of complex systems^17–21^. In particular, scale-free networks, in which the degree distribution follows a power law *P*(*k*) ∝ *k*^−*γ*^ with an exponent *γ*, can model various systems with scaling properties such as citation patterns in science^22^, internet^23^, e-mail connections^24^, to name but a few. Scale-free networks display a variety of interesting transport features found in nature^23,25^. In particular, the global point-to-point flux Φ in a random scale-free resistor network between two arbitrarily chosen points was shown to obey a distribution with a power law tail: *P*(Φ) ∝ Φ^−(2*γ*−1)^ ^26,27^. The degree exponent *γ* was linked to the scaling of the flux in the line-to-line transport in a resistor network with multiple sources and drains^28^.

In a typical setting, a particle, an animal, a disease, a virus, a toxin, a signal or a rumor is released at one location and then spreads over the network. To get a theoretical insight onto this phenomenon, a first step is to consider the *point-to-point transport* between two arbitrary nodes, treated as a source and a drain. In this paper, we investigate the electric transport in a class of scale-free resistor networks, obeying Kirchhoff’s laws or, equivalently, a Markov chain random walk on weighted graphs. This study reveals the structural organization of the nodes potentials and currents in the links. It is found that the density of currents, *p*(*ϕ*), decays at large *ϕ* as a power law with the universal exponent −2. This behavior is attributed to the arborescent structure of links that drive the currents from the source to the drain through a large *quasi-equipotential (QEP) cluster* of nodes. In other words, the transport between two selected nodes of a network is governed by two random trees, for which the exponent −2 can be justified by theoretical arguments.

## Results

A random scale-free network is constructed on an *N* × *N* square lattice. Its links are generated by using an uncorrelated configuration model^29^ with a given degree exponent *γ*. To get more insight onto the universal character of the point-to-point transport, we consider the resistance *r*_*i*,*j*_ of each link as a function of the Euclidean distance *d*_*i*,*j*_ between the nodes *i* and *j*: ${r}_{i,j}={d}_{i,j}^{\beta }$, with an exponent *β*. In an electric or hydraulic circuit, the resistance of a wire or a tube is proportional to its length, and *β* = 1. In turn, most former studies on transport in resistor networks supposed constant link resistance, i.e., *β* = 0 (see, e.g.^26^). In each random realization of the resistor network with prescribed exponents *γ* and *β*, we select randomly a source node and a drain node, at which the potential is fixed to be 1 and 0, respectively. The system of linear Kirchhoff’s equations^30^ for the potential on other nodes is solved numerically using a custom routine in Matlab (see Methods section). Then the distributions of nodes potentials and currents in the links are obtained. While we keep using the terminology of electric circuits, the results are valid for diffusive and hydraulic transport as well.

### Universal power-law distribution of currents

**Figure 1:**
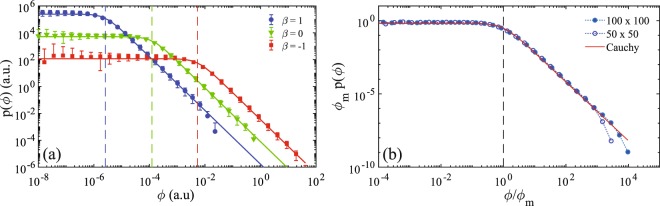
(**a**) The density of currents in links of a scale-free resistor network on a lattice 100 × 100, with *γ* = 3 and three values of *β*. The empirical probability densities were first estimated from a set of currents in each random realization of the network and then averaged over 300 realizations. Symbols show the average values while errorbars indicate the average plus or minus the standard deviation. The bottom part of the errorbar is missing whenever the average minus the standard deviation is negative. The one-sided Cauchy density in Eq. (1) with the median currents from Table 1 is shown by solid lines. Dashed vertical lines indicate the median currents *ϕ*_*m*_ for each *β*. Similar results were obtained for other values of *γ* (not shown). (**b**) The empirical density of currents rescaled by the median current *ϕ*_*m*_ for a scale-free resistor network with *γ* = 3, *β* = 1, *N* = 100 (full circles) and *N* = 50 (empty circles), as compared to the one-sided Cauchy density (solid line). Here, the empirical probability densities were obtained by merging local currents from 300 random realizations of the network to improve the statistics (errorbars are not available here).

The first striking result is that the distribution of currents in a random scale-free resistor network follows a universal power law distribution, that is independent of the degree distribution exponent *γ* and the resistance-distance exponent *β*. Figure 1(a) shows that the density of currents is constant at small currents and then decays as a power law with the exponent −2. This probability density admits an excellent fit by the one-sided Cauchy distribution (for *ϕ* ≥ 0),

1 $$p(\varphi )=\frac{2}{\pi }\frac{{\varphi }_{m}}{{\varphi }_{m}^{2}+{\varphi }^{2}},$$

where *ϕ*_*m*_ is the median current. Interestingly, the mean and the variance of this distribution are infinite for an infinite network, which is a reminiscent property of scale-free systems. The universal power law decay can potentially be related to formerly studied betweenness measures in scale-free networks^31–33^. While the distribution is universal, the median current *ϕ*_*m*_ depends on the properties of the network (Table 1). The median current *ϕ*_*m*_ has a relatively weak dependence on *γ*, as compared to substantial changes of the properties of the scale-free network for various *γ*. The much stronger dependence on *β* results from a significant change of the length-dependent resistances: when *β* = 1, resistances are proportional to inter-node distances and are thus large (yielding small currents); in turn, for *β* = −1, the resistances are much smaller while the currents are larger. The deviations from the power law decay *p*(*ϕ*) ∝ *ϕ*^−2^ at large currents can be attributed to a finite size of the system. In fact, for any finite-size resistor network, the inter-node distances are bounded from above and below, and thus there exists a minimal resistance and a maximal current. As a consequence, the distribution of currents has a finite support bounded by a maximal cut-off. The cut-off value depends on the exponent *β* and the size *N* of the system, and increases as *N* → ∞. This is clearly seen on Fig. 1(b), which shows the density of currents rescaled by the median current *ϕ*_*m*_ for *N* = 50 and *N* = 100. The rescaling is needed to make closer the densities for two cases because the median current depends on the system size. One can see that the empirical density for the larger system remains close to the theoretical curve up to larger currents, i.e., the cut-off current is larger.

**Table 1:** The median current *ϕ*_*m*_ for different exponents *γ* and *β*.

*γ*\*β*	1	0	−1
1.5	0.56	0.27	1.26
2.0	1.21	0.58	2.48
2.5	2.02	0.92	4.14
3.0	2.40	1.16	5.00
4.0	2.99	1.52	5.77
5.0	3.52	1.73	6.58
6.0	3.74	1.91	6.95
	×10^−6^	×10^−4^	×10^−3^

For each value of *γ* and *β*, the empirical distribution was obtained by merging currents from 300 random realizations of scale-free networks on a lattice 100 × 100.

### Morphological organization of the point-to-point transport

**Figure 2:**
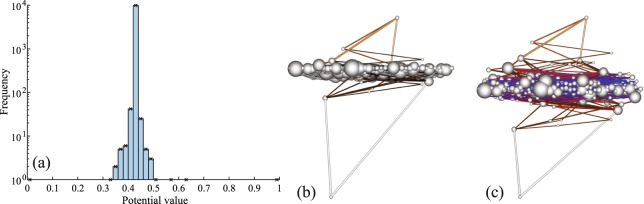
(**a**) Histogram of the nodes potentials (lattice 100 × 100, *β* = 1, and *γ* = 2.5). One observes that the overwhelming majority of nodes have almost identical potential. (**b**,**c**) Visualization of a point-to-point transport on a scale-free network (with a lattice 40 × 40, *γ* = 2.5 and *β* = 1; high resolution images are available online). Each node of the network is shown by a ball whose radius is proportional to the square root of its connectivity. The planar coordinates of the balls are the positions of the corresponding nodes on the square lattice, whereas the height *Z* is related to the potential at the node, either by a linear relation *Z* = *V* (**b**) or by a nonlinear relation *Z* ∝ s*ign*(*V* − *V*_*m*_)|*V* − *V*_*m*_|^1/2^ (**c**), where *V*_*m*_ is the mode of the potential histogram, and rescaling is used to ensure that the bottom black ball at *Z* = 0 is the drain at fixed potential 0 and the top white ball at *Z* = 1 is the source at fixed potential 1. Such a nonlinear relation helps to dilate the QEP cluster to visualize its structure. Each link brightness is proportional to the magnitude of its current (in addition, blue colors are used for very small currents in panel (c)). The QEP cluster is qualitatively identified as a large ensemble of nodes almost at the same potential.

To get a deeper insight onto the above transport properties, we study the distribution of the nodes potentials. By construction, the potential varies between 0 (the drain) to 1 (the source). Figure 2(a) shows an example of the potential distribution for one realization of the scale-free network with *γ* = 2.5 and *β* = 1. The most striking feature is the very peaked distribution of potentials around a particular value (here, 0.43). In other words, the overwhelming majority of nodes share very close potentials and form thus a quasi-equipotential cluster, which works as an almost perfect conductor. This analysis gives a more precise description of the so-called transport backbone^26^. While the particular value of the potential of the QEP cluster is specific to the network realization, the very strong concentration around this particular value is universal, being observed for other values of *γ* and *β*.

The structural organization of the point-to-point transport is visualized in Fig. 2(b,c). Here the nodes of a scale-free network are elevated in the vertical direction according to their potential. One can distinguish the QEP cluster (the region in the middle), and two arborescent structures, one rooted in the source at the top, and the other rooted in the drain at the bottom. The “terminal branches” of both trees are connected to the QEP cluster and can thus be considered as grounded to the potential of the QEP cluster. This structural organization allows one to treat the point-to-point transport on networks as a series connection of two random trees. As the electric properties of a tree can be determined via exact recursive computations (see below), this is a tremendous simplification of the original problem. We will show how this discovery brings new conceptual understanding of the point-to-point transport on networks and a rational for the observed universality in the currents distribution. The almost flat region of the density of currents in Fig. 1 can be attributed to the small currents in links between the numerous nodes and loops of the QEP cluster. In turn, the larger currents flow in two trees (see Fig. 2(b,c)) and produce a power law decay of the currents distribution. This is typical for a tree, in which the largest current in the trunk is progressively divided in a large number of smaller currents following the successive branching points. As both trees are connected to the QEP cluster, they can be treated independently.

### Recursive computation for a tree

**Figure 3:**
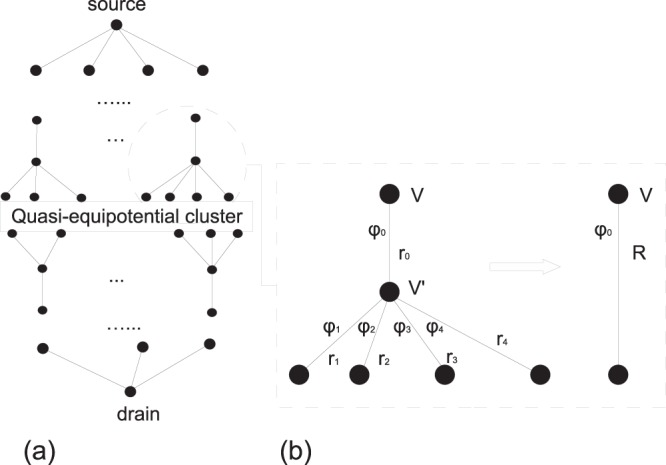
(**a**) Schematic representation of the two-sided arborescent structure created by applying potentials to two points: one tree is rooted in the drain at the bottom and includes nodes with distinct potentials, and the second random tree is rooted in the source on the top and also includes nodes with distinct potentials. The “terminal branches” of both trees are connected to the QEP cluster. If the latter is substituted by an equipotential one, the currents can be computed exactly by a recursive procedure. (**b**) Each step of the recursive procedure consists in substituting a group of nodes with resistances *r*_0_, *r*_1_, …, *r*_*m*_ by an effective link with the overall resistance *R*. Repeating this step, one evaluates the total resistance and then all intermediate potentials and currents.

For an arbitrary resistors tree, in which all terminal branches are grounded (set to a potential *V*_0_) and a given potential *V*_1_ is applied to the root, the currents and potentials can be computed via an exact recursive procedure. In fact, for any terminal branch, one can identify its mother node and all sister nodes (Fig. 3). If *r*_1_, …, *r*_*m*_ denote the resistances of the links from these terminal branches to their mother node, and *r*_0_ is the resistance of the link from the mother node to its mother node, then the overall resistance of this group is simply *R* = *r*_0_ + 1/(1/*r*_1_ + … + 1/*r*_*m*_). As a consequence, this group of links can be replaced by a single effective link with the resistance *R*. Repeating this replacement procedure from the most distant terminal branches and progressing toward the root, we can compute the total resistance of the tree *R*_tree_, from which we get the local current (*V*_1_ − *V*_0_)/*R*_tree_ at an effective link representing the whole tree, and the potential drops at the daughter nodes. Considering now each daughter node as the root of the corresponding subtree, one can repeat the computation. Descending recursively from the root to the most distant terminal branches, one evaluates all currents and potentials.

**Figure 4:**
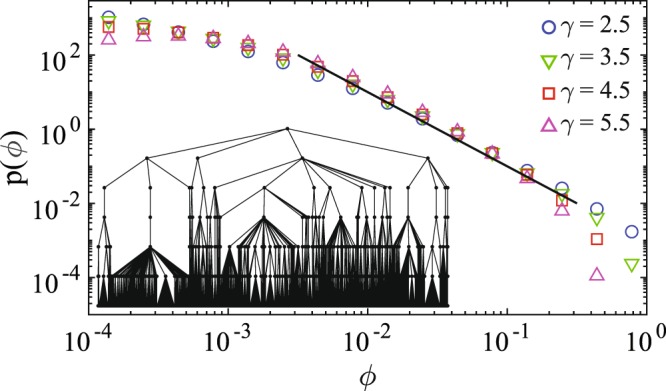
The density of currents in random trees built by choosing randomly the node degrees from the degree distribution for several *γ* values (within a 100 × 100 lattice). To keep the total number of nodes in the trees in a range between 1000 and 3000, the number of generations *G* (i.e., the distance from the root to any terminal node), was assigned differently for different *γ*: *G* = 7 (*γ* = 2.5), *G* = 12 (*γ* = 3.5), *G* = 19 (*γ* = 4.5), and *G* = 27 (*γ* = 5.5). Currents were computed by fixing the potential to 1 at the source and 0 at the the last generation nodes. All branches have unit resistance (i.e., *β* = 0). The density is obtained from a histogram of currents from 300 realizations. Irrespective of *γ* values, the density of currents decays as a power law with an exponent −2, indicated with the straight line (the power law is terminated by a cut-off due to a finite size of the tree). The inset shows an example of a random tree with *γ* = 2.5.

At any level, once the potential *V* at the mother of the mother node is evaluated for a given group, we can compute the current *ϕ*_0_ = *V*/*R*, the potential

$$V^{\prime} =V-{\varphi }_{0}{r}_{0}=V(1-{r}_{0}/R)=\frac{V}{1+\frac{{r}_{0}}{{r}_{1}}+\ldots +\frac{{r}_{0}}{{r}_{m}}},$$

and the currents *ϕ*_*i*_ = *V*′/*r*_*i*_ at each link. If the resistances *r*_*i*_ are comparable to each other, then the currents *ϕ*_*i*_ in the links to daughter nodes are approximately *m* + 1 times smaller than the current *ϕ*_0_, while the number of such currents is *m* times larger. Most importantly, due to the arborescent character of the structure, this reduction of currents is repeated multiplicatively at all branching levels. Therefore, the probability that the local current $\hat{\varphi }$ at a randomly chosen link exceeds a prescribed value *ϕ*, is dominated by the relative fraction of links with the current of order *ϕ*, which is inversely proportional to *ϕ*. In other words, ${\mathbb{P}}\{\hat{\varphi }\ge \varphi \}\propto 1/\varphi$, from which the scaling *ϕ*^−2^ of the probability density *p*(*ϕ*) follows immediately. These qualitative arguments are confirmed by a more rigorous computation for some classes of random trees (see Methods section) and by numerical simulations for various random trees (see Fig. 4).

## Discussion

The emergence of a “working tree” in a complex network is not a surprise since the currents follow the links that were built independently of the choice of source and drain. Starting for the source node, there exits a finite number of links leading to other nodes. At this stage there are no loops. Then starting from these new nodes, one finds new links but the probability of creating loops is very small because the great majority of the support sites are free for new links. This process builds progressively a tree structure up to the situation where loops are necessarily created when the number of tree sites is comparable to total number of support sites. This is why the numerical simulations shown in Fig. 4 have been limited to a total number of tree nodes between 1/10 and 1/3 of the total number of sites of the support lattice.

While our discussion was focused on electric currents and potentials, the discovered morphological organization of point-to-point transport is relevant for other phenomena. For instance, steady-state diffusion of particles from a source to a sink is governed by the Laplace equation whose discretization on a given graph yields a set of linear equations similar to the Kirchhoff’s equations. In this setting, electric potentials and currents are substituted by concentrations of particles and their fluxes, respectively. Moreover, when the source concentration is set to 1, concentration at a given point can alternatively be interpreted as the splitting probability that a particle started from that point hits the source point before hitting the drain point. In other words, it characterizes the relative “accessibilities” of a source and a drain from different points of the graph^34^.

In summary, the application of a potential difference at two randomly chosen points of a scale-free network determines a transport structure composed of two random trees (rooted at these two points) connected through a quasi-equipotential cluster. Small currents between the nodes of this cluster form the plateau region of the density of currents. The arborescent character of the resistant part of this structure implies the multiplicative reduction of the currents in these trees and thus a power law decay of the density of currents with the universal exponent −2. Whatever the rules of construction of a scale-free network (e.g. the choice of *β* and *γ*), the density of currents exhibits the same decay. Note that similar structural organizations of the point-to-point transport are found in various natural and human-engineered complex systems: human vascular network (with an artery splitting progressively into numerous capillaries and then merging again to few veins), braided river networks^35,36^, water supply networks and irrigation systems (with one or several water intake stations supplying a branching network of pipes and then merging again to one or several sewage disposal points), to name but a few. Here, we showed that such a structural organization is formed spontaneously in a scale-free network by applying the potential difference to two arbitrary points. This observation is expected to help to understand the morphology, optimality, and robustness of natural point-to-point transport systems. An extension of this study to other types of complex networks such as small-world graphs, presents an interesting perspective for future research. More generally, our interpretation in terms of arborescent structure of links that drive the currents from source to drain through a large QEP cluster of nodes opens the door to aggregate conceptualizations of transport processes in complex networks.

## Methods

### Description of the numerical scheme

Numerical simulations of the scale-free resistor networks are performed using MATLAB 9.2 (R2017a). The source (potential set to 1) and drain (potential set to 0) nodes are randomly picked among nodes that are separated by a distance of at least 4 nodes. The system of linear Kirchhoff’s equations^30^, excluding the source and drain nodes, reads in a matrix form as *M* × *P* = *S*, where *P* is the column vector of all *N* − 2 unknown potentials, *M* is the (*N* − 2) × (*N* − 2) matrix of coefficients, and *S* is the column vector of *N* − 2 elements, where each element corresponds to the total flow exiting the current node *j* and satisfying the conservation of mass equation. The system is solved using the typical “mldivide” (or backslash) operator in MATLAB, that computes the solution *P* using LU decomposition. Knowing the potential *P*, one can then compute the current for each link.

### Computation of current distribution on a tree

We discuss two examples of trees for illustrating the general arguments of the main text about the distribution of currents.

First, we consider a regular tree with *N* levels of branching, in which each node (except the root) is divided into *q* daughter branches. We select the root node as the source and all terminal nodes as a drain. The correspondence to the point-to-point transport can be made by merging this tree to its copy (“reflected tree”) by connecting pairwise all terminal nodes. In that case, the root of the reflected tree is set as the drain. The distribution of currents is the same in both cases due to the symmetry. As this network is deterministic, the currents are as well deterministic, so that one can understand the distribution of currents in terms of frequencies of observation of a given value of the current. Clearly, such a distribution is discrete.

Due to the symmetry, currents in all branches of a given level are identical. If *ϕ*_0_ denotes the current in the root branch (of level 0), which is single by construction, then the current in any branch of the level *n* is just *ϕ*_0_/*q*^*n*^, where *q*^*n*^ is the number of branches at this level. Then the probability (interpreted as the frequency of occurrence) for the current $\hat{\varphi }$ in a randomly selected branch is

2 $${P}_{n}={\mathbb{P}}\{\hat{\varphi }={\varphi }_{0}/{q}^{n}\}=\frac{{q}^{n}}{1+q+\ldots +{q}^{N-1}}\,,$$

from which, denoting *ϕ* = *ϕ*_0_/*q*^*n*^, one has

3 $${\mathbb{P}}\{\hat{\varphi }\ge \varphi \}={P}_{0}+{P}_{1}+\ldots +{P}_{n}=\frac{q{\varphi }_{0}/\varphi -1}{{q}^{N}-1}.$$

Formally treating *ϕ* as a continuous variable, one finds that the corresponding probability density function bahaves as

4 $$p(\varphi )=-\frac{\partial {\mathbb{P}}\{\hat{\varphi }\ge \varphi \}}{\partial \varphi }\propto \frac{1}{{\varphi }^{2}}.$$

This computation can be extended a nonregular tree, in which the number *q*_*n*_ of daughter branches depends on the branching level *n*. Repeating the above arguments, one gets

5 $${\mathbb{P}}\{\hat{\varphi }\ge \varphi \}=A(1+{q}_{1}+{q}_{1}{q}_{2}+\ldots +{q}_{1}{q}_{2}\cdots {q}_{n}),$$

where *ϕ* = *ϕ*_0_/(*q*_1_*q*_2_ … *q*_*n*_), and *A* = 1 + *q*_1_ + *q*_1_*q*_2_ + … + *q*_1_*q*_2_ … *q*_*N*−1_ is the normalization constant. Since all *q*_*n*_ ≥ 2, one can easily prove that the last term, *q*_1_*q*_2_ … *q*_*n*_, provides the dominant contribution to this sum. In other words, the contribution of the remaining terms is either smaller or comparable to the last term. In fact, the following inequality holds

6 $$1+{q}_{1}+{q}_{1}{q}_{2}+\ldots +{q}_{1}{q}_{2}\cdots {q}_{n-1}\le {q}_{1}{q}_{2}\cdots {q}_{n}.$$

To prove it, one can divide both sides by *q*_1_*q*_2_ … *q*_*n*−1_ and check that

$$\frac{1}{{q}_{1}{q}_{2}\cdots {q}_{n-1}}+\ldots +\frac{1}{{q}_{n-1}}+1\le \frac{1}{{2}^{n-1}}+\ldots +\frac{1}{2}+1=2(1-{(1/2)}^{n})\le 2\le {q}_{n}.$$

As a consequence, ${\mathbb{P}}\{\hat{\varphi }\ge \varphi \}\propto 1/\varphi$, and thus one gets again the scaling exponent −2 for the current density.

## Data Availability

Data sharing not applicable to this article as no datasets were generated or analysed during the current study.
